# Adaptive Planning Method for ERS Point Layout in Aircraft Assembly Driven by Physics-Based Data-Driven Surrogate Model

**DOI:** 10.3390/s26030955

**Published:** 2026-02-02

**Authors:** Shuqiang Xu, Xiang Huang, Shuanggao Li, Guoyi Hou

**Affiliations:** College of Mechanical and Electrical Engineering, Nanjing University of Aeronautics and Astronautics, Nanjing 210016, China; xushuqiang168@nuaa.edu.cn (S.X.); lishuanggao@nuaa.edu.cn (S.L.); hou_gy@nuaa.edu.cn (G.H.)

**Keywords:** aircraft assembly, laser tracker, enhanced reference system (ERS), coordinate transformation uncertainty, BP neural network

## Abstract

In digital-measurement-assisted assembly of large aircraft components, the spatial layout of Enhanced Reference System (ERS) points determines coordinate transformation accuracy and stability. To address manual layout limitations—specifically low efficiency, occlusion susceptibility, and physical deployment limitations—this paper proposes an adaptive planning method under engineering constraints. First, based on the Guide to the Expression of Uncertainty in Measurement (GUM) and weighted least squares, an analytical transformation sensitivity model is constructed. Subsequently, a multi-scale sample library generated via Monte Carlo sampling trains a high-precision BP neural network surrogate model, enabling millisecond-level sensitivity prediction. Combining this with ray-tracing occlusion detection, a weighted genetic algorithm optimizes transformation sensitivity, spatial uniformity, and station distance within feasible ground and tooling regions. Experimental results indicate that the method effectively avoids occlusion. Specifically, the Registration-Induced Error (RIE) is controlled at approximately 0.002 mm, and the Registration-Induced Loss Ratio (RILR) is maintained at about 10%. Crucially, comparative verification reveals an RIE reduction of approximately 40% compared to a feasible uniform baseline, proving that physics-based data-driven optimization yields superior accuracy over intuitive geometric distribution. By ensuring strict adherence to engineering constraints, this method offers a reliable solution that significantly enhances measurement reliability, providing solid theoretical support for automated digital twin construction.

## 1. Introduction

With the shift towards digitalization in aviation manufacturing, large-scale metrology systems [1,2,3], particularly laser trackers [4,5], have become indispensable. However, due to the immense size of aircraft components, a single instrument is often limited by its field of view, necessitating the construction of a distributed measurement network [6,7,8]. In this network, the Enhanced Reference System (ERS) point is critical for unifying measurement data into the assembly coordinate system (ACS), directly determining the final assembly quality [9,10]. Unfortunately, constructing an efficient ERS point network remains a challenge. Key factors such as station selection and point layout [11] significantly impact the solution stability [12,13]. Traditional planning relies heavily on engineer experience, suffering from ineffective integration of complex constraints like occlusion [14] and low efficiency due to repeated on-site trial and error [15].

Regarding coordinate transformation uncertainty, a robust theoretical framework has been established. Scholars have analyzed error propagation mechanisms using Weighted Total Least Squares (WTLS) [16], Hybrid Reference Systems [17,18,19], and multi-system coupling models [20,21]. These studies, grounded in the GUM [22] and matrix decomposition algorithms [23,24,25], provide solid mathematical support. On this basis, layout optimization has become a research hotspot. Strategies focusing on sensitivity coefficients [26], feature-oriented configurations [27], and instrument accessibility [28,29] have been proposed to improve transformation accuracy.

Gap Analysis and Proposed Method Despite these advancements, the rise of digital twin technology [30,31,32] has imposed new demands for measurement systems to possess self-perception and adaptive capabilities [33,34]. However, achieving this with traditional analytical methods is computationally prohibitive when handling complex constraints. While recent attempts utilizing genetic algorithms and neural networks [35,36] or deep learning [37,38] have improved efficiency, they often lack deep integration with physical transformation mechanisms. Furthermore, mathematically optimal points generated by current algorithms often “lack physical support”, rendering them impractical for deployment.

In view of this, this paper proposes an adaptive planning method for ERS point layout driven by a physics-based data-driven surrogate model (a BP neural network trained on GUM-generated labels). Here, ‘physics-based’ refers to the GUM-driven uncertainty propagation used for label generation, rather than embedding governing equations into the loss function/architecture as in PINN-type approaches. First, a physical model of coordinate transformation sensitivity is established based on GUM theory; second, a sensitivity rapid prediction surrogate model based on a BP neural network is constructed to replace time-consuming analytical calculations; finally, combined with ray-tracing occlusion detection and an improved genetic algorithm, an ERS layout scheme that meets accuracy constraints with the minimum number of points is adaptively searched for in the digital twin scenario. This method aims to solve the trial-and-error problem of traditional manual station deployment and provide an efficient and reliable solution for the automated construction of high-precision measurement fields in aircraft assembly.

## 2. Rapid Prediction Method for ERS Transformation Matrix Sensitivity Based on GUM-Weighted Sensitivity Model and BP Neural Network

**Figure 1:**
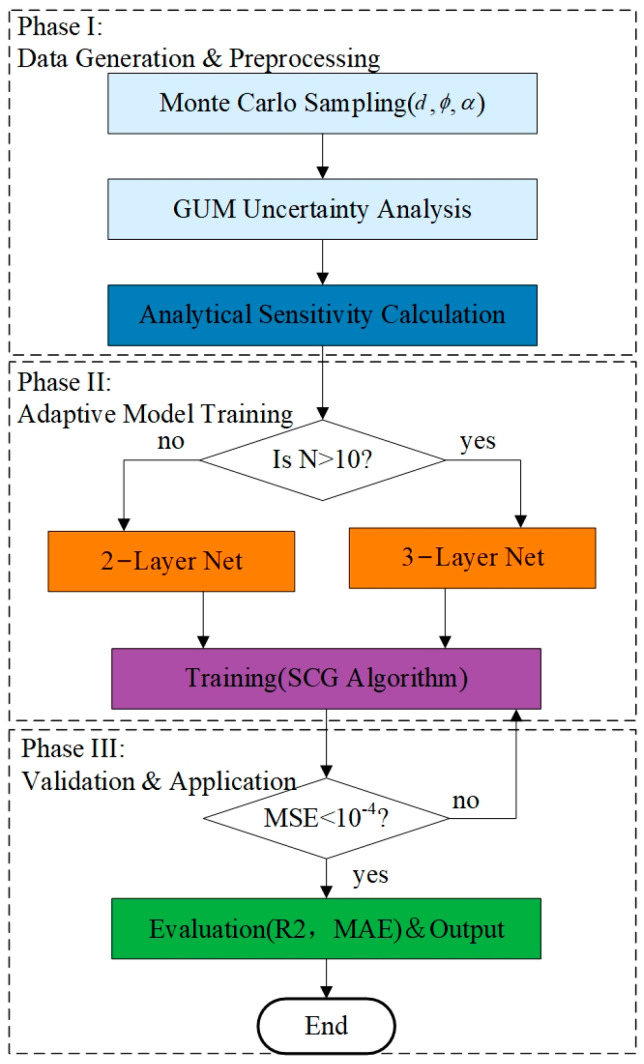
Methodological workflow for rapid prediction of transformation sensitivity based on GUM and BP neural network.

To achieve rapid optimization of reference point configurations in large-scale aircraft assembly, this paper constructs a unified methodological framework that synergizes “physically interpretable sensitivity modeling” with “data-driven rapid prediction,” as illustrated in Figure 1. The framework proceeds as follows: First, based on the GUM theory, the single-point measurement covariance is derived and propagated into a weighted least squares model to rigorously quantify the transformation parameter sensitivity index (*S*). Serving as the physics engine, this analytical model generates a comprehensive library of multi-scale configuration samples (*N* = 4~15). Subsequently, these samples are utilized to train a BP neural network, enabling it to learn the complex non-linear mapping between “geometric layout” and “sensitivity performance,” thereby achieving millisecond-level prediction with high fidelity.

### 2.1. Uncertainty Analysis of Laser Tracker Measurement

#### 2.1.1. Laser Tracker Measurement Uncertainty Modeling

Spatial coordinates of a point by measuring the radial distance d, azimuth angle ϕ, and zenith angle α of the target point relative to the instrument center, based on the instrument coordinate system {*M*}. To use Cartesian coordinates in subsequent registration and sensitivity analysis, the spherical coordinates $\left(d,\phi ,\alpha \right)$ need to be converted to Cartesian coordinates $\left(x,y,z\right)$. The conversion relationship is expressed as:

(1) $$\left[\begin{array}{c} x\\ y\\ z \end{array}\right]=\left[\begin{array}{c} d\sin\alpha \cos\phi \\ d\sin\alpha \sin\phi \\ d\cos\alpha \end{array}\right]$$

where *d* ≥ 1 m, $\phi \in \left[0^{\circ },360^{\circ }\right)$, $\alpha \in \left[45^{\circ },135^{\circ }\right]$. These angles apply only to the Leica AT901-MR (Leica Geosystems AG, Heerbrugg, Switzerland), which is the measurement instrument selected in this paper.

**Figure 2:**
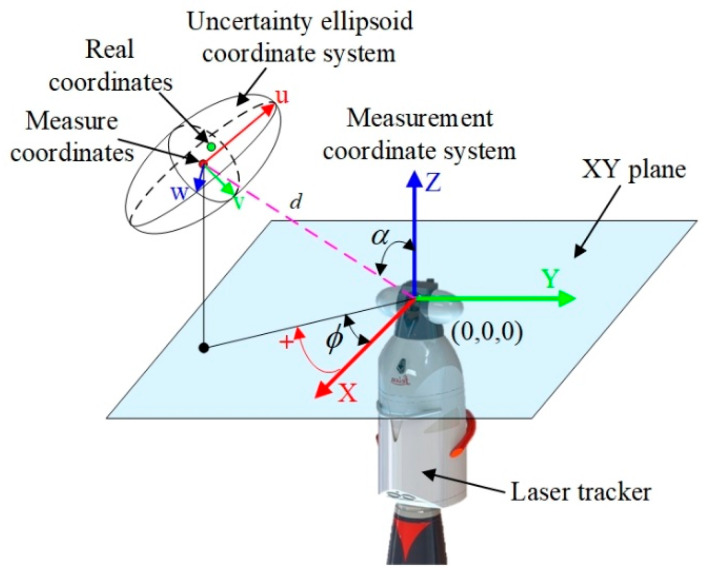
Coordinate definition and uncertainty ellipsoid model of the laser tracker.

Due to inherent noise in the laser tracker sensors, measurement values inevitably contain random errors. The uncertainty sources mainly include the Interferometer/Absolute Distance Meter (IFM/ADM), the angular encoders, and environmental disturbances (temperature, humidity, atmospheric pressure) causing changes in the refractive index of air. Typically, laser trackers are equipped with temperature, humidity, and pressure sensors to correct the atmospheric refractive index in real time. Therefore, the contribution of environmental disturbances to coordinate uncertainty can be neglected, and only the contribution of the measured values from the instrument’s sensing units needs to be considered. Parameters such as distance d, azimuth ϕ, and zenith all inevitably contain uncertainty, leading to coordinate measurement uncertainty. In 3D space, this uncertainty can be visualized as an uncertainty ellipsoid, as shown in Figure 2.

To quantify this error, this paper adopts the method recommended by the Guide to the Expression of Uncertainty in Measurement (GUM) for modeling. Since the measurement errors of the ranging sensor (IFM/ADM) and the angular encoders follow a normal distribution and the components are independent of each other, their error covariance matrix in the spherical coordinate system is a diagonal matrix:

(2) $$\sum_{sph}=diag\left(\sigma_{d}^{2},\sigma_{\phi }^{2},\sigma_{\alpha }^{2}\right)$$

where

$\sigma_{d}$: Distance uncertainty, usually including system error and proportional error, expressed as:

(3) $$\sigma_{d}=7.62\text{μm}+2.5\text{μm}\times L$$

where

*L* is the measured distance in meters.

$\sigma_{\phi }$ and $\sigma_{\alpha }$: Angular uncertainty, converted from the instrument’s angular resolution. The angular uncertainty is 1 arc second:

(4) $$\sigma_{\phi }=\sigma_{\alpha }=1^{\prime \prime }=\frac{\pi }{180\times 3600}rad$$

Using the law of propagation of error, by performing a first-order Taylor expansion on Equation (1), the measurement uncertainty covariance matrix $\sum_{xyz}$ of the target point in the Cartesian coordinate system can be obtained:

(5) $$\sum_{xyz}=J\cdot \sum_{sph}\cdot J^{T}$$

where *J* is the Jacobian Matrix of the coordinate transformation, defined and derived as follows:

(6) $$J=\left[\begin{array}{ccc} \frac{\partial x}{\partial d} & \frac{\partial x}{\partial \phi } & \frac{\partial x}{\partial \alpha }\\ \frac{\partial y}{\partial d} & \frac{\partial y}{\partial \phi } & \frac{\partial y}{\partial \alpha }\\ \frac{\partial z}{\partial d} & \frac{\partial z}{\partial \phi } & \frac{\partial z}{\partial \alpha } \end{array}\right]=\left[\begin{array}{ccc} \sin\alpha \cos\phi & -d\sin\alpha \sin\phi & d\cos\alpha \cos\phi \\ \sin\alpha \sin\phi & d\sin\alpha \cos\phi & d\cos\alpha \sin\phi \\ \cos\alpha & 0 & -d\sin\alpha \end{array}\right]$$

Thus, $\sum_{xyz}$, which reflects the anisotropic measurement characteristics, can be obtained for each reference point, providing precise data basis for the subsequent evaluation of transformation parameter sensitivity.

#### 2.1.2. Calculation of Transformation Parameter Sensitivity Based on Covariance Matrix

Quality of coordinate transformation depends not only on the measurement accuracy of single points but also on the spatial configuration of the reference point group. To quantify the impact of reference point distribution on the stability of the transformation matrix solution, this paper establishes a sensitivity solution model more consistent with actual working conditions, based on the ERS point configuration analysis theory proposed in Reference [26] and introducing the anisotropic covariance matrix $\sum_{xyz}$.

Before measuring, the laser tracker needs to calculate the rigid body transformation parameters from the instrument coordinate system {*M*} to the assembly coordinate system {*A*} using a group of common ERS points. Let the measured coordinates of the *i*-th common reference point in {*M*} be $P_{i}^{M}$ and its nominal coordinates in {*A*} be $P_{i}^{A}$. The two satisfy the rigid body transformation relationship:

(7) $$P_{i}^{A}=RP_{i}^{M}+T+\varepsilon_{i}$$

where *R* is the rotation matrix, *T* is the translation vector, and $\varepsilon_{i}$ is the measurement residual. For a measurement field containing *N* common ERS points, the coordinate transformation problem is usually converted into an objective function minimizing the weighted registration error. Let *q* be the transformation parameter error vector to be solved, containing three small rotation error components $\left(\delta \alpha ,\delta \beta ,\delta \gamma \right)$ and three translation error components $\left(\delta t_{x},\delta t_{y},\delta t_{z}\right)$:

(8) $$q={\left[\delta \alpha ,\delta \beta ,\delta \gamma ,\delta t_{x},\delta t_{y},\delta t_{z}\right]}^{T}$$

According to the linearized error equation, the reference point measurement error and the transformation parameter error satisfy a linear relationship:

(9) Cq=e

where $e\in {\mathbb{R}}^{3N}$ is the Cartesian coordinate measurement error vector of all points, and $C\in {\mathbb{R}}^{3N\times 6}$ is the configuration matrix of the reference point group, which is entirely determined by the geometric distribution of the ERS points in the global coordinate system. *C* is defined as:

(10) $$C_{i}=\left[\begin{array}{cccccc} 0 & z_{i} & -y_{i} & 1 & 0 & 0\\ -z_{i} & 0 & x_{i} & 0 & 1 & 0\\ y_{i} & -x_{i} & 0 & 0 & 0 & 1 \end{array}\right],C=\left[\begin{array}{c} C_{1}\\ C_{2}\\ \vdots \\ C_{N} \end{array}\right]$$

where $\left(x_{i},y_{i},z_{i}\right)$ are the nominal coordinates of the *i*-th common reference point.

Considering that the measurement uncertainty $\sum_{xyz,i}$ of different common ERS points differs significantly (heteroscedasticity), the traditional least squares method is no longer applicable. This paper uses the weighted least squares (WLS) method for optimal estimation to obtain the posterior covariance matrix of the transformation parameters. The weight matrix *W* is defined as a block diagonal matrix composed of the inverse matrices of the measurement error covariance matrices:

(11) $$W=diag\left(\sum_{xyz,1}^{-1},\sum_{xyz,2}^{-1},\cdots \sum_{xyz,N}^{-1}\right)\in {\mathbb{R}}^{3N\times 3N}$$

According to the generalized least squares principle, the covariance matrix $\sum_{q}={\mathbb{R}}^{6\times 6}$ of the transformation parameter error vector can be obtained via the inverse of the information matrix *H*:

(12) $$H=C^{T}WC$$

(13) $$\sum_{q}={\left(C^{T}WC\right)}^{-1}=\left[\begin{array}{ccc} K_{\alpha } & \ldots & \ldots \\ \vdots & \ddots & \vdots \\ \ldots & \ldots & K_{z} \end{array}\right]$$

(14) $$diag\left(\sum_{q}\right)=\left[K_{\alpha },K_{\beta },K_{\gamma },K_{x},K_{y},K_{z}\right]$$

The first three terms of $diag\left(\sum_{q}\right)$ correspond to the estimation variances of the three-axis rotation parameters, and the last three terms correspond to the estimation variances of the three-axis translation parameters. The smaller these values are, the stronger the suppression capability of the current reference point configuration against measurement noise, and the more stable the calculated transformation parameters.

To evaluate the overall configuration quality with a single scalar, the comprehensive sensitivity index *S* is defined as the trace of $\sum_{q}$:

(15) $$S=tr\left(\sum_{q}\right)=K_{\alpha }+K_{\beta }+K_{\gamma }+K_{x}+K_{y}+K_{z}$$

The value of *S* is the core target value that the BP neural network in this paper needs to predict. Using the trace of the covariance matrix as the comprehensive evaluation index implies pursuing A-optimality, aiming to minimize the average value of the transformation parameter estimation variances.

#### 2.1.3. Multi-Scale Training Sample Construction

To enable the neural network to learn the complex non-linear mapping from “geometric distribution” to “transformation sensitivity,” constructing a training sample set that is widely covered, randomly distributed, and large-scale is crucial.

(1) Sample Scale and Dimension Design

In actual large-scale aircraft component assembly scenarios, the number of common points used for coordinate transformation is usually more than four (although three points satisfy the mathematical minimum, they lack redundancy and are rarely used in engineering), and the computational benefit diminishes while operation becomes cumbersome after exceeding 15 points. Therefore, this paper sets the range for the number of ERS points as *N* ∈ [4~15].

Dataset generation employs Monte Carlo sampling to simulate diverse configurations. For each *n*, the sample size is stratified: *M* = 50,000 when *N* ≤ 10 (dense coverage for low dimensions); *M* = 10,000 when *N* > 10 (to address high-dimensional sparsity).

For each *N*, this paper adopts a stratified massive sampling strategy to generate $M_{N}$ sets of ERS geometric and uncertainty samples:

(16) $$M_{N}=\left\{\begin{array}{c} 5\times 10^{4},N\leq 10\\ 1\times 10^{5},N>10 \end{array}\right.$$

(2) Sample Generation Process

The Monte Carlo method is used to randomly generate samples within the measurement space. For a fixed *N*, the steps for generating a single sample are:

1. Random Sampling: Within the laser tracker’s measurement space (*d* ∈ [4~15 m]), $\phi \in \left[0^{\circ },360^{\circ }\right)$, $\alpha \in \left[45^{\circ },135^{\circ }\right]$), the k-th sample is defined as:
  (17) $$x_{k}^{\left(N\right)}=\left[d_{1},\phi_{1},\alpha_{1},d_{2},\phi_{2},\alpha_{2},\cdots ,d_{N},\phi_{N},\alpha_{N}\right]\in {\mathbb{R}}^{3N}$$
  where $k=1,2,\ldots ,M_{N}$. To eliminate ambiguity caused by ordering, sort points within each sample set first by distance d and then by ϕ, in descending order.
2. Forced Validity: Ensure the minimum distance between points is >300 mm. This simulates the physical constraint that points cannot overlap in actual deployment, avoiding collinearity or cluster degradation.
3. Data Augmentation: Introduce 2% “bad” samples (clustered angles) to improve robustness.
4. Uncertainty Calculation: Calculate the measurement uncertainty covariance matrix $\sum_{xyz}$ for each point according to Equation (5).
5. Sensitivity Solution: Construct the configuration matrix *C* and weight matrix W, and calculate the transformation parameter error vector covariance matrix $\sum_{q}$ using Equation (12).
6. Feature Extraction: Extract the diagonal elements $\left[K_{\alpha },K_{\beta },K_{\gamma },K_{x},K_{y},K_{z}\right]$ of $\sum_{q}$ and calculate the trace to obtain the sensitivity label for this sample:
  (18) $$y_{k}^{\left(N\right)}=\left[K_{\alpha },K_{\beta },K_{\gamma },K_{x},K_{y},K_{z},S\right]\in {\mathbb{R}}^{7}$$

(3) Sample Storage Format

To facilitate neural network reading and training, the generated sample data is standardized and stored in matrix form. For any number (*N*) of ERS points, its mathematical expression is:

(19) $$D^{\left(N\right)}={\left\{x_{k}^{\left(N\right)},y_{k}^{\left(N\right)}\right\}}_{k=1}^{M_{N}}\in {\mathbb{R}}^{M_{N}}$$

This storage method provides a clear data interface for subsequent input/output disassembly, standardization, and model management. Thus, this paper completes the full-link construction from instrument measurement uncertainty to the covariance-weighted sensitivity index and then to the multi-scale training sample library, laying the data foundation for the subsequent rapid prediction by the BP neural network.

### 2.2. BP Neural Network Data Processing

#### 2.2.1. Data Partitioning and Input/Output Sample Definition

From Section 2.1, for a fixed *n*, the input matrix and output matrix are respectively:

(20) $$X^{\left(N\right)}={\left[x_{1}^{\left(N\right)},\ldots ,x_{M_{N}}^{\left(N\right)}\right]}^{T}\in {\mathbb{R}}^{M_{N}\times 3N}$$

(21) $$Y^{\left(N\right)}={\left[y_{1}^{\left(N\right)},\ldots ,y_{M_{N}}^{\left(N\right)}\right]}^{T}\in {\mathbb{R}}^{M_{N}\times 7}$$

To ensure statistical fairness in training and evaluation, the samples are randomly divided into a training set, validation set, and test set. Three sample index sets are introduced:

(22) $$I_{train},I_{val},I_{test}\subset \left\{1,2,\ldots ,M\right\}$$

They satisfy mutual disjointedness, their union is the entire sample set, and they follow a ratio of 70%/15%/15%, used for model parameter learning, generalization monitoring, and final performance evaluation. The corresponding sample numbers are:

(23) $$M_{train}=\left|I_{train}\right|,M_{val}=\left|I_{val}\right|,M_{test}=\left|I_{test}\right|$$

(24) $$M=M_{train}+M_{val}+M_{test}$$

#### 2.2.2. Z-Score Standardization of Input Data

Since distance d, azimuth ϕ, and zenith α have different value ranges and dimensions, directly inputting raw features into the neural network would lead to significant differences in numerical scale, thereby reducing the efficiency of gradient descent. To improve training stability and convergence speed, Z-score standardization is performed column-wise on the input matrix $X^{\left(N\right)}$.

For the j-th feature dimension $\left(j=1,\ldots ,3N\right)$, let its sample mean and standard deviation on the training set be:

(25) $$\mu_{j}=\frac{1}{M_{train}}\sum_{k\in I_{train}}x_{k,j},\sigma_{j}=\sqrt{\frac{1}{M_{train}}\sum_{k\in I_{train}}{\left(x_{k,j}-\mu_{j}\right)}^{2}}$$

Then, the standardized feature is:

(26) $${\tilde{x}}_{k,j}=\frac{x_{k,j}-\mu_{j}}{\sigma_{j}}$$

The standardized matrix is denoted as:

(27) $${\tilde{X}}^{\left(N\right)}=\left[{\tilde{x}}_{k,j}\right]\in {\mathbb{R}}^{M_{N}\times 3N}$$

This transformation normalizes each column to zero mean and unit standard deviation, eliminating scale differences between dimensions while preserving the relative relationships between original geometric features, conducive to synchronized convergence of gradient-based optimization algorithms across dimensions.

#### 2.2.3. Logarithmic Transformation and Normalization of Output Labels

**Figure 3:**
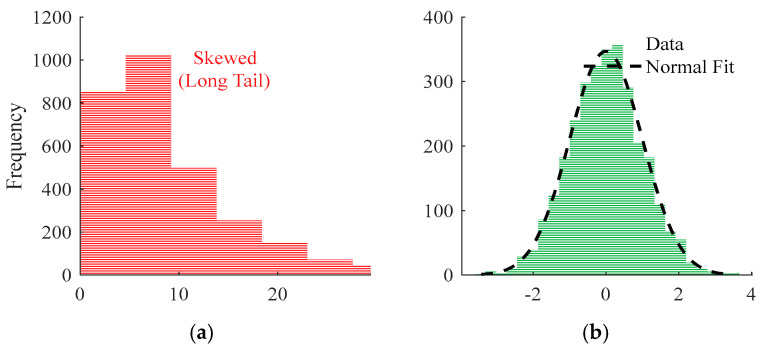
Comparison of sensitivity index (S) distribution before and after log-normalization. (**a**) Sensitivity index S (raw); (**b**) processed S’ (log-norm).

Since the values of the sensitivity parameter and the comprehensive index S span multiple orders of magnitude, the output distribution under different geometric configurations often exhibits long-tail characteristics. To avoid a small number of maximum samples dominating the loss function and weakening the network’s learning ability in the low-to-medium sensitivity range, this paper adopts a logarithmic domain reconstruction strategy for the output. The intuitive effect of the improved distribution is shown in Figure 3.

The element-wise logarithmic transformation for each sample’s output vector $y_{k}^{\left(N\right)}$ is denoted as:

(28) $$z_{k}^{\left(N\right)}=\log_{10}\left(y_{k}^{\left(N\right)}\right)$$

The log-domain output matrix is obtained as:

(29) $$Z^{\left(N\right)}={\left[z_{1}^{\left(N\right)},\ldots ,z_{M_{N}}^{\left(N\right)}\right]}^{T}\in {\mathbb{R}}^{M_{N}\times 7}$$

Then, for each output dimension $\left(r=1,\ldots ,7\right)$:

(30) $${\hat{z}}_{k,r}=2\frac{z_{k,r}-z_{r}^{\min}}{z_{r}^{\max}-z_{r}^{\min}}-1$$

where $z_{r}^{\min}$ and $z_{r}^{\max}$ are obtained from the training set statistics. The normalized output matrix is denoted as:

(31) $${\hat{Z}}^{\left(N\right)}=\left[{\hat{z}}_{k,r}\right]\in {\mathbb{R}}^{M_{N}\times 7}$$

As shown in Figure 3a, the original comprehensive sensitivity index S exhibits a significant right-skewed and long-tailed distribution in physical space; Figure 3b shows the distribution of $S^{\prime }$ after logarithmic transformation, and normalization is more concentrated and approximately symmetrical. The logarithmic transformation significantly weakens the long-tailed effect of the original sensitivity index, making the output distribution more suitable for regression training based on the mean squared error. Therefore, this step is a key data processing step for achieving cross-configuration stable prediction.

### 2.3. BP Neural Network Model and Training Algorithm

**Figure 4:**
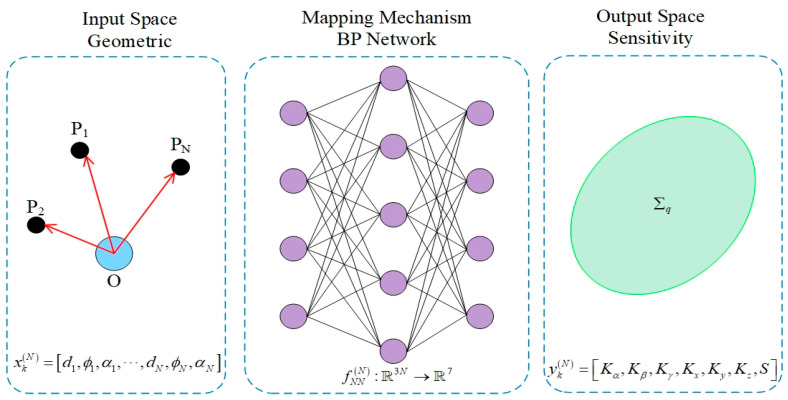
Architecture of the BP neural network mapping geometric features to sensitivity indices.

After completing input/output definition and log-domain data reconstruction, this paper further constructs different BP neural network regression models for different N to learn the statistical mapping relationship between “geometric observation–sensitivity index”. The overall framework is shown in Figure 4.

The mapping framework transforms traditional sensitivity analysis computation into fast inference of neural networks, enabling sensitivity evaluation of a large number of candidate ERS point configurations with extremely low computational cost, laying the methodological foundation for subsequent ERS point layout screening and optimization iteration.

#### 2.3.1. Forward Propagation Model

For a fixed number of ERS points N, a multi-layer feedforward BP neural network is constructed with $\tilde{x}\in {\mathbb{R}}^{3N}$ as input and $\hat{z}\in {\mathbb{R}}^{7}$ as output. Let the network contain L hidden layers, with $W^{\left(l\right)}$ and $b^{\left(l\right)}$ as the weight matrix and bias vector of the l-th layer, respectively, and the L+1-th layer as the output layer, where l=1,…,L+1.

The forward propagation formulas are:

(32) $$\begin{array}{l} h^{\left(0\right)}=\tilde{x}\\ a^{\left(l\right)}=W^{\left(l\right)}h^{\left(l-1\right)}+b^{\left(l\right)},l=1,\ldots ,L+1\\ h^{\left(l\right)}=\left\{\begin{array}{c} \sigma \left(a^{\left(l\right)}\right),l=1,\ldots ,L\\ a^{\left(l+1\right)},l=L+1 \end{array}\right. \end{array}$$

where $\sigma \left(\cdot \right)$ is the non-linear activation function. The hidden layers use the Tansig function, and the output layer uses the Purelin function. The network output is:

(33) $$\hat{z}=h^{\left(L+1\right)}$$

#### 2.3.2. Loss Function and Objective Optimization

Given a training sample set $D_{train}^{\left(N\right)}$, the training objective of a BP network is to minimize the mean squared error (MSE) between the predicted output and the target output. Let ${\hat{z}}_{k}^{\left(tar\right)}$ be the target output of the k-th training sample and ${\hat{z}}_{k}^{\left(pred\right)}$ be the network prediction. Then, the loss function is defined as:

(34) $$J\left(\Theta \right)=\frac{1}{M_{N}^{train}}{\sum_{k\in I_{train}}\left\|{\hat{z}}_{k}^{\left(pred\right)}-{\hat{z}}_{k}^{\left(tar\right)}\right\|}_{2}^{2}$$

where Θ represents all network weights and bias parameters. This loss function is equivalent to a least-squares fit to the logarithmic domain sensitivity index, which physically corresponds to minimizing the relative sensitivity error.

#### 2.3.3. Back Propagation and Gradient Calculation

The BP algorithm recursively calculates the gradient of the loss function with respect to the parameters of each layer using the chain rule. For the output layer:

(35) $$\delta_{k}^{\left(L+1\right)}=\frac{\partial J}{\partial a_{k}^{\left(L+1\right)}}={\hat{z}}_{k}^{\left(pred\right)}-{\hat{z}}_{k}^{\left(tar\right)}$$

For the l-th hidden layer $\left(l=1,\ldots ,L\right)$, we have:

(36) $$\delta_{k}^{\left(l\right)}={\left(W^{\left(l+1\right)}\right)}^{T}\delta_{k}^{\left(l+1\right)}⊙\sigma^{\prime }\left(a_{k}^{\left(l\right)}\right)$$

where ⊙ denotes element-wise multiplication, and $\sigma^{\prime }$ is the derivative of the activation function. The gradient of the parameters in each layer can then be obtained:

(37) $$\frac{\partial J}{\partial W^{\left(l\right)}}=\frac{1}{M_{N}^{train}}\sum_{k\in I_{train}}\delta_{k}^{\left(l\right)}{\left(h_{k}^{\left(l-1\right)}\right)}^{T}$$

(38) $$\frac{\partial J}{\partial b^{\left(l\right)}}=\frac{1}{M_{N}^{train}}\sum_{k\in I_{train}}\delta_{k}^{\left(l\right)}$$

These gradients serve as input to subsequent optimization algorithms (such as conjugate gradients) to iteratively update network parameters.

#### 2.3.4. Training Algorithm

Traditional backpropagation (BP) typically employs a gradient-descent-based weight update strategy, where convergence speed is highly dependent on the step size. To improve training efficiency on large-scale datasets, this paper uses the scaled conjugate gradient (SCG) algorithm for iterative parameter updates.

In the t-th iteration, SCG approximately constructs a quadratic model in the current search direction $d_{t}$:

(39) $$J\left(\Theta_{t}+\eta d_{t}\right)\approx J\left(\Theta_{t}\right)+\eta g_{t}^{T}d_{t}+\frac{1}{2}\eta^{2}d_{t}^{T}B_{t}d_{t}$$

where $g_{t}=\nabla J\left(\Theta \right)$ and $B_{t}$ are approximations of the Hessian. $d_{t}^{T}B_{t}d_{t}$ can be estimated through one or two additional forward–backward propagations, thereby analytically obtaining the optimal step size $\eta_{t}$ and updating the parameters:

(40) $$\Theta_{t+1}=\Theta_{t}+\eta_{t}d_{t}$$

SCG, while maintaining the advantages of conjugate gradient directions, avoids the overhead of an explicit line search, making it suitable for acceleration on GPUs using large-scale matrix operations. Combined with the aforementioned Z-score and log-normalized preprocessing, the network can still converge stably on 5 × 10^4^~10^5^-scale samples.

### 2.4. Dynamic Network Structure Design and Hyperparameter Settings

**Figure 5:**
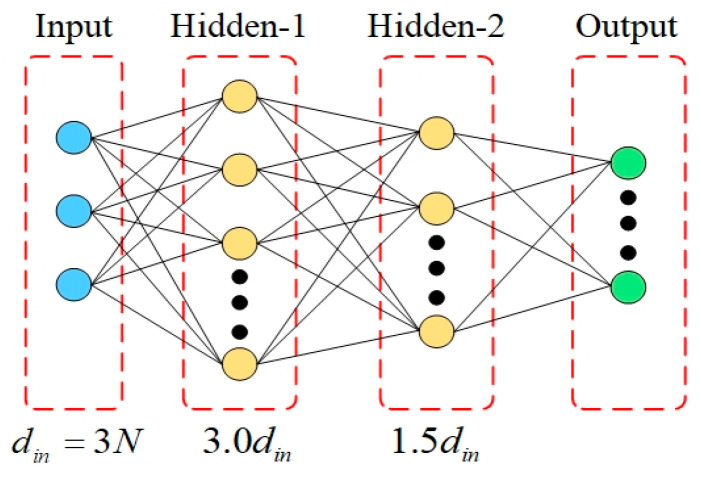
Adaptive network architecture shallow structure with two hidden layers.

**Figure 6:**
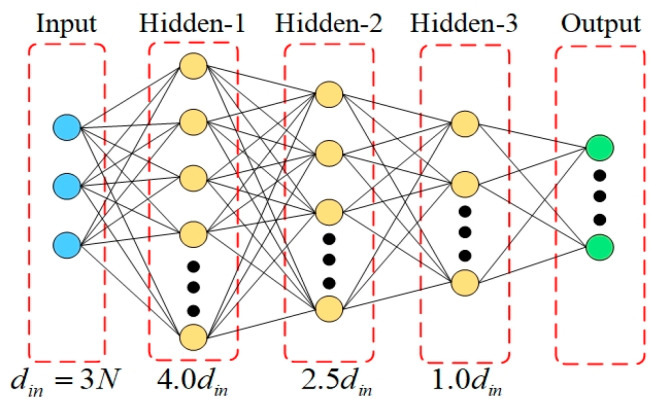
Adaptive network architecture deep structure with three hidden layers.

As the number of ERS points N increases, the input dimension 3N grows linearly, and the complexity of geometric configuration and the non-linearity of sensitivity response increase synchronously. Using a fixed-scale network makes it difficult to balance the fitting needs of low-dimensional and high-dimensional problems. Therefore, this paper proposes a dynamic network structure design based on input dimension, as shown in Figure 5 and Figure 6.

Let the input dimension be:

(41) $$d_{in}=3N$$

When N≤10, a two-hidden-layer structure is adopted, as shown in Figure 5:

(42) $$l_{1}=\left\lceil 3.0d_{in}\right\rceil ,l_{2}=\left\lceil 1.5d_{in}\right\rceil$$

Note that the ceiling function $\left\lceil \cdot \right\rceil$ is applied to ensure the number of neurons is an integer when $d_{in}$ is odd.

When N>10, a three-hidden-layer structure is adopted, as shown in Figure 6:

(43) $$l_{1}=\left\lceil 4.0d_{in}\right\rceil ,l_{2}=\left\lceil 2.5d_{in}\right\rceil ,l_{3}=\left\lceil 1.0d_{in}\right\rceil$$

As shown in the figures, the adaptive switching between shallow and deep layers provides matching model capacity for problems of different dimensions. To determine the optimal switching threshold, a comparative ablation study was conducted. The results indicated that for low-dimensional inputs (*N* ≤ 8), a two-hidden-layer structure achieves lower mean squared error (MSE) and faster convergence compared to deeper architectures, which tend to suffer from overfitting in this regime. However, as the dimension increases (*N* ≥ 10), the two-layer network exhibits signs of under-fitting. Consequently, the architecture switches to a three-hidden-layer structure for high-dimensional scenarios, where the increased depth significantly improves the model’s capacity to capture complex non-linear features (reducing MSE by over 6.0% at *N* = 12). This dynamic design ensures high-precision sensitivity prediction across the entire range of N while maintaining computational efficiency.

### 2.5. Model Performance Evaluation and Sensitivity Prediction Result Verification

#### 2.5.1. Training Phase Performance

During model training, the dataset is randomly partitioned into three disjoint subsets: training, validation, and test. The training subset is dedicated to updating the network weights via backpropagation. The validation subset serves to monitor the generalization error for early stopping and model selection (i.e., preventing overfitting) and is strictly excluded from the gradient update process. The test subset is held out completely during the training phase and is utilized only afterwards to evaluate the model’s internal generalization performance under the same data-generation distribution. To quantify the regression performance, the correlation coefficient (*R*) and the mean squared error (MSE) are adopted as the primary metrics. The correlation coefficient *R* measures the linear correlation between the target sensitivity values (calculated by GUM) and the output values (predicted by the BP network), where an *R* value close to 1 denotes a strong positive correlation.

Table 1 summarizes the network training performance metrics for different numbers of ERS points, including Train R, Val R, Test R, All R, and the optimal validation MSE. It can be seen that, at N = 4, the model already provides a usable fit. As N increases, the correlation coefficient rapidly increases and then stabilizes at a high level.

Especially in the *N* = 11~15 range, all *R* values are above 0.99, and the optimal MSE is approximately on the order of 1.1 × 10^−3^. This indicates that the data preprocessing and dynamic structure design in this paper can effectively support high-dimensional sensitive mapping learning.

**Figure 7:**
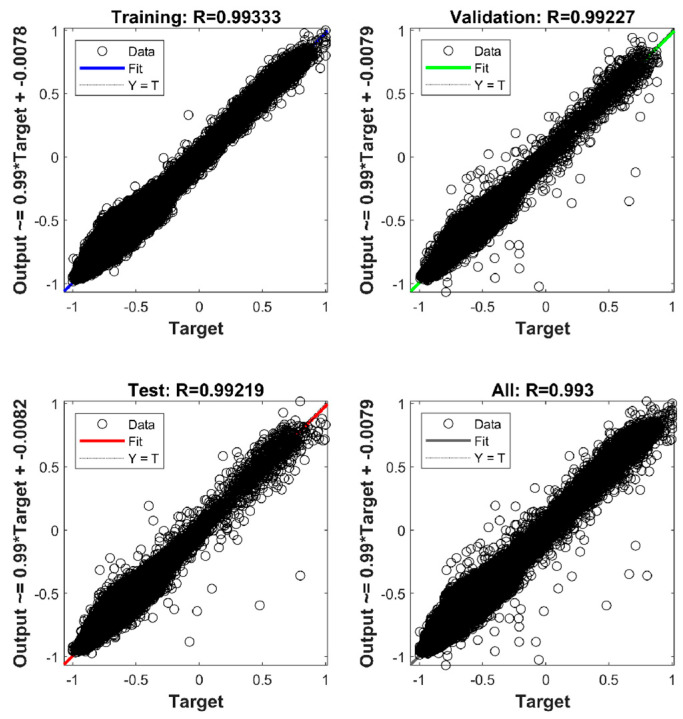
Regression analysis between the network outputs and physics-based targets on training/validation/test subsets (the reported value is the correlation coefficient *R*).

Taking *N* = 11 as an example, the regression plot of the BP surrogate model is shown in Figure 7. This figure illustrates the linear correlation between the network predictions and the physics-based targets (GUM-generated labels). Specifically, Figure 7 reports the correlation coefficient *R* (as returned by the MATLAB(R2024a) regression plot) for the training, validation, and test subsets, which are $R_{train}=0.993$, $R_{val}=0.992$ and $R_{test}=0.992$, respectively, with an overall $R_{all}=0.993$. These results indicate a strong agreement between predicted and target values across all subsets.

**Figure 8:**
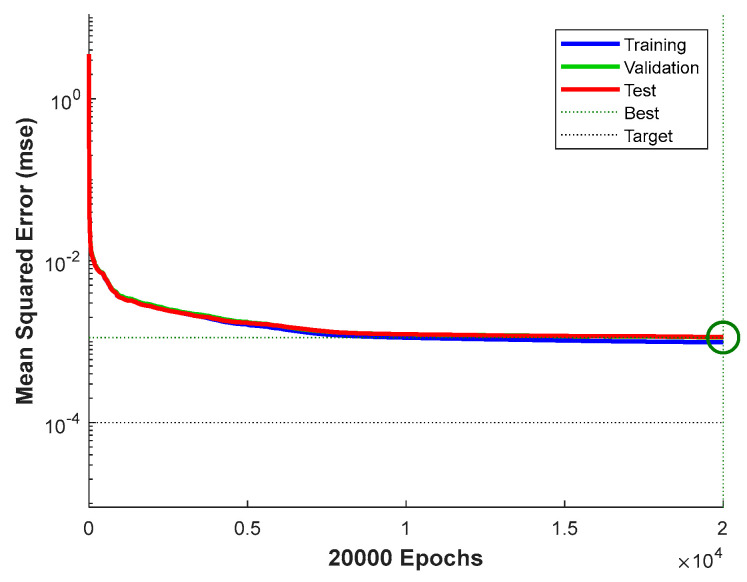
Training convergence curve: mean squared error (MSE) performance over epochs.

The training process curve in Figure 8 shows that the best validation performance occurred in the 20,000th round, with the MSE dropping to 0.0011261, and the training curve converged smoothly, verifying the effectiveness of the SCG algorithm and the convergence and effectiveness of the model.

#### 2.5.2. Independent Validation Set and Evaluation Metrics

To further test the network’s generalization ability on unseen configurations, a new set of $M_{N}^{ver}=1000$ baseline configurations was randomly generated for each N using the Monte Carlo method. Under each configuration, the “true value” sensitivity vector $Y_{k,phys}^{\left(N\right)}$ was calculated using the physical model in Section 2.1, and the predicted value ${\hat{Y}}_{k,phys}^{\left(N\right)}$ was obtained using the trained BP network.

Regarding evaluation metrics, considering the numerous output dimensions and the consistent dimensions of each component, this paper uses the comprehensive index *S* as a representative for detailed analysis. Let:

(44) $$y_{k}=\log_{10}S_{k},{\hat{y}}_{k}=\log_{10}{\hat{S}}_{k}$$

Then, in the logarithmic field, the coefficient of determination $R_{\log}^{2}$ is defined as:

(45) $$R_{\log}^{2}=1-\frac{\sum_{k=1}^{M_{N}^{ver}}{\left(y_{k}-{\hat{y}}_{k}\right)}^{2}}{\sum_{k=1}^{M_{N}^{ver}}{\left(y_{k}-\bar{y}\right)}^{2}},\bar{y}=\frac{1}{M_{N}^{ver}}\sum_{k=1}^{M_{N}^{ver}}y_{k}$$

The root mean square error in the logarithmic field is:

(46) $$RMSE_{\log}=\sqrt{\frac{1}{M_{N}^{ver}}}\sum_{k=1}^{M_{N}^{ver}}{\left(y_{k}-{\hat{y}}_{k}\right)}^{2}$$

The absolute error of the physical domain is:

(47) $$e_{i}^{phys}=\left|S_{i}-{\hat{S}}_{i}\right|$$

The corresponding mean absolute error is:

(48) $$MAE_{phys}=\frac{1}{M_{N}^{ver}}\sum_{k=1}^{M_{N}^{ver}}\left|S_{k}-{\hat{S}}_{k}\right|$$

The median relative error is:

(49) $$MRE_{phys}=median\left(\frac{\left|S_{i}-{\hat{S}}_{i}\right|}{\left|S_{i}\right|+\varepsilon }\right)$$

The 95th percentile absolute error is:

(50) $$P95_{phys}=percentile_{95}\left(e_{i}^{phys}\right)$$

#### 2.5.3. Verification Results Analysis

The overall prediction accuracy analysis and verification results are summarized in Table 2. The model demonstrates excellent generalization ability, with an average coefficient of determination of 0.9876 for all test configurations and an average relative error of only 3.79%. This indicates that the model can not only accurately predict the order of magnitude of sensitivity (logarithmic domain) but also maintain extremely high fidelity after being restored to physical values.

**Figure 9:**
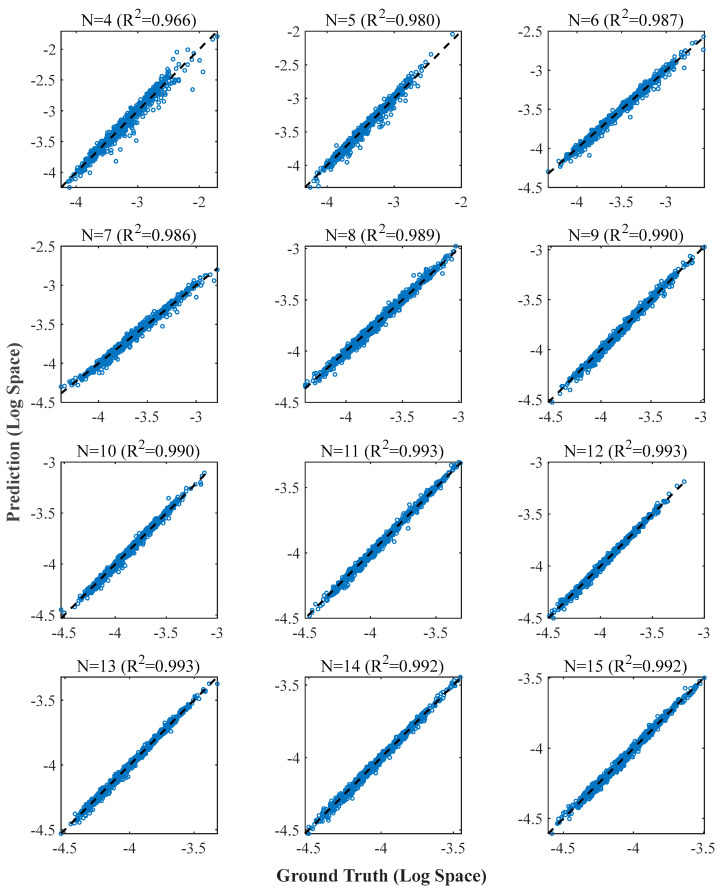
Log-space prediction accuracy verification across multi-scale configurations.

Figure 9 shows the comparison between the predicted and actual values of the independent validation set in log space. It can be seen that as the number of ERS points N increases, the data points converge more tightly to the diagonal of y=x. When N≥5, the $R^{2}$ value exceeds 0.98, demonstrating that the model has robust predictive ability for sensitivity indices at different scales.

**Figure 10:**
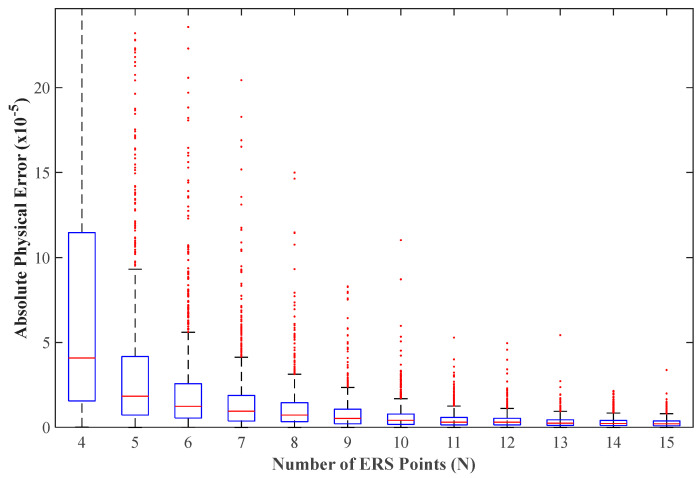
Distribution of absolute physical prediction errors under different ERS point quantities.

Figure 10 further reveals the distribution pattern of the absolute error in physical space. The box plot clearly shows that as the number of ERS points N increases, the median and dispersion of the prediction error decrease significantly. When N≥11, the physical prediction error of the vast majority of samples is controlled within the order of 5 × 10^−5^ or even smaller, with only very few configurations showing slightly larger deviations. This trend is consistent with the analytical sensitivity findings in Section 2.1, that is, redundant observations can improve the stability of the configuration matrix and enhance the ability to suppress measurement noise.

Notably, for typical engineering applications (e.g., N=11), the model’s mean absolute error (MAE) is reduced to the level of 4.50 × 10^−6^ mm, and the median relative error (MRE) is controlled at 2.64%. More importantly, the P95 metric, representing the model’s reliability boundary, shows that 95% of the test samples have a prediction error of less than 1.28 × 10^−5^ mm. For N=15, P95 is even lower, reaching 8.45 × 10^−6^ mm.

Meanwhile, the computation speed of the BP neural network model was also statistically analyzed. Taking N=11 as an example, the number of samples M was 100, 1000, 10,000, and 100,000 respectively. The statistical results are shown in Table 3. It can be seen that the larger the number of combinations, the greater the improvement in the computational efficiency of the BP surrogate model. When *M* = 10^5^, the GUM sensitivity calculation took 7.85 s, while the BP surrogate model only took 0.21 s, which improved the computational efficiency by 37 times. This is only a single operation. During the baseline layout planning process, a large number of samples and multiple iterations will be generated, and the BP surrogate model has a clear advantage in computational efficiency.

In summary, the independent validation results demonstrate from three aspects—trend consistency, average error, and upper bound of high quantile—that the BP prediction model in this paper can stably reconstruct the comprehensive sensitivity S in the multi-scale ERS configuration space of N=4~15, providing a reliable and efficient surrogate model support for subsequent rapid selection of ERS points and layout optimization based on sensitivity criteria.

## 3. ERS Point Layout Planning Method Based on Transformation Sensitivity Surrogate Model

**Figure 11:**
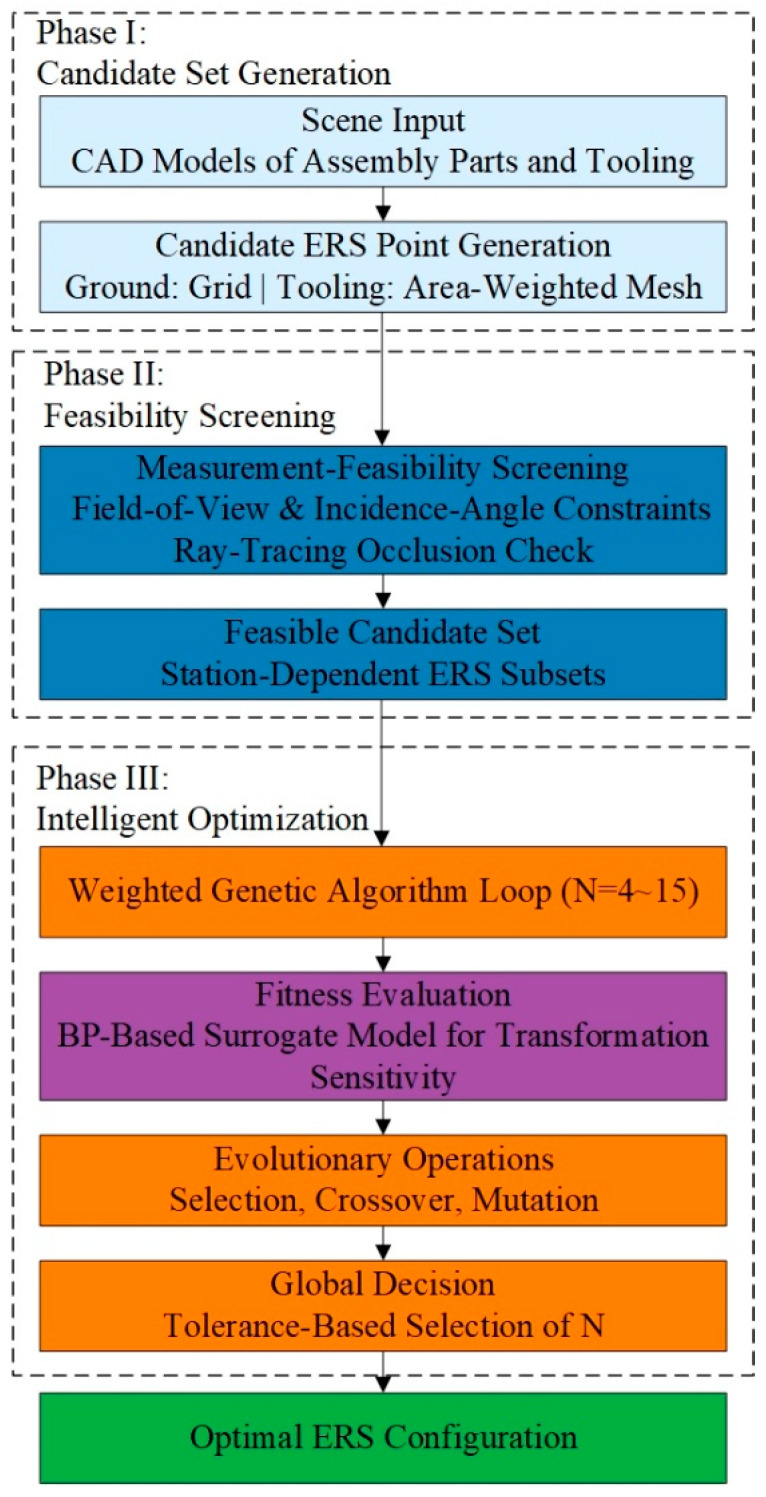
Flowchart of ERS point layout planning strategy driven by BP neural network surrogate model.

Building upon the sensitivity surrogate model established in Section 2, this section proposes an adaptive ERS layout planning method tailored to specific aircraft assembly sites. This method prioritizes the sensitivity index ($S$) as the primary objective while incorporating spatial uniformity and station-distance preferences as auxiliary constraints. As illustrated in Figure 11, the planning workflow is structured into three integrated phases: (1) feasibility screening: generating candidate points on tooling/ground surfaces and filtering them via FOV and occlusion checks; (2) intelligent optimization: employing a BP-driven weighted genetic algorithm to explore the optimal geometric configuration; and (3) global decision: applying a point-number adaptive mechanism to automatically identify the minimal layout scheme that satisfies stringent accuracy requirements.

### 3.1. Candidate Reference Point Set Generation

#### 3.1.1. Ground Candidate Point Generation

In large aircraft assembly workshops, some ERS points are typically located on or near the ground on foundation structures. Ground ERS points offer engineering advantages such as simple installation and long-term stability and can be shared by multiple measurement stations. Therefore, this paper first generates a set of high-density ground candidate points on a virtual ground plane.

Considering the relative height relationship between the assembly fixture and the ground, in the simulation, this paper uses the minimum z-coordinate $z_{\min}^{tool}$ of all nodes of the assembly fixture model as the virtual ground height $z_{gnd}$, that is:

(51) $$z_{gnd}=\underset{k}{\min}\left(z_{k}^{tool}\right)$$

A two-dimensional regular grid is constructed on this plane according to the spacing Δx=Δy=100 mm:

(52) $$x_{i}\in \left[x_{\min}-M,x_{\max}+M\right],y_{i}\in \left[y_{\min}-M,y_{\max}+M\right]$$

where $\left[x_{\min},x_{\max}\right]$ and $\left[y_{\min},y_{\max}\right]$ are determined by the envelope of the fuselage, wings, and tooling model, and M is the outward expansion dimension. For each mesh node $\left(x_{i},y_{i}\right)$, a ground candidate point is defined:

(53) $$P_{k}^{GND}={\left(x_{i},y_{i},z_{gnd}\right)}^{T}$$

It is assigned a unique identifier ID, for example, to generate ground candidate points that not only spatially cover the entire assembly area but also can be easily implemented in the actual workshop through ground markers or supports.

#### 3.1.2. Tooling Surface Candidate Point Generation

While relying solely on ground-based ERS points offers advantages, the distance between the ERS points and the target points is typically significant, potentially amplifying measurement noise due to the transformation matrix. Therefore, this paper further arranges candidate ERS points on the surfaces of the fuselage and wing fixtures, making the ERS points more spatially aligned with the measured components.

Specifically, the STL meshes of the fuselage and wing fixtures are randomly sampled based on area, followed by voxel mesh filtering for down-sampling and homogenization. Assuming the fixture mesh consists of a set of triangular facets, with each facet having an area of $A_{m}$, the initial number of samples can be determined based on the total area and the desired sampling density ρ.

(54) $$N_{raw}=\left|\rho \sum_{m}A_{m}\right|$$

For each sampling point, its three-dimensional coordinates $P_{n}^{raw}$ are generated according to the principle of uniform sampling within the triangular face. These coordinates are then projected onto a voxel mesh and clustered by voxel size. Each voxel retains at most one representative point, resulting in a uniform set of tooling candidate points $\left\{P_{i}^{Tool}\right\}$ and $\left\{P_{j}^{Tool}\right\}$. The identifiers ID for each point are “FG_JIG_k “for the fuselage tooling reference point or “WG_JIG_k” for the wing tooling reference point, facilitating differentiation of their origin.

#### 3.1.3. Candidate Reference Point Set Data Structure and Naming Convention

Based on the above steps, the candidate ERS point set constructed in this paper can be uniformly represented as:

(55) $$\Omega =\left\{\left(P_{k},ID_{k},Type_{k}\right)|k=1,\ldots ,K\right\}$$

where $P_{k}=\left(x_{k},y_{k},z_{k}\right)$ represents the three-dimensional coordinates of the candidate reference point set, $ID_{k}$ is a unique string identifier, and $Type_{k}\in \left\{Ground,Tooling\right\}$ indicates its source category.

### 3.2. Candidate Reference Point Set Screening

#### 3.2.1. Instrument FOV and Angle Constraints

Given a measurement station s and a candidate point $P_{k}$, denoted as $M^{\left(s\right)}$, and the *Z*-axis of the instrument coordinate system as $u_{z}^{\left(s\right)}$, the measurement ray vector is:

(56) $$r_{k}^{\left(s\right)}=P_{k}-M^{\left(s\right)}$$

The zenith angle can be determined by:

(57) $$\cos\alpha_{k}^{\left(s\right)}=\frac{r_{k}^{\left(s\right)}\cdot u_{z}^{\left(s\right)}}{\left\|r_{k}^{\left(s\right)}\right\|}$$

The calculation shows that if point $\alpha_{k}^{\left(s\right)}$ exceeds the instrument’s specified field of view, the candidate point is directly determined as unmeasurable at station s and is therefore eliminated. This constraint reflects the limitations of the instrument’s physical structure and drive mechanism and is unrelated to sensitivity indicators.

#### 3.2.2. Measurement LOS Occlusion Check

While meeting the instrument’s field-of-view requirements, it is still necessary to check whether the measurement ray is obstructed by the fuselage, wings, or tooling structures. Therefore, this paper adopts a ray tracing strategy of “axis-aligned bounding box (AABB) coarse screening + precise intersection of triangular faces + parallel computation”. Specifically, for each measurement station s and candidate point $P_{k}$, denoted as measurement station $M^{s}$, a ray is constructed from $M^{s}$ to $P_{k}$:

(58) $$x\left(t\right)=M^{s}+td_{m}^{\left(s\right)},t\in \left[0,l_{k}^{\left(s\right)}\right]$$

where distance is $l_{k}^{\left(s\right)}=\left\|r_{k}^{\left(s\right)}\right\|$, unit direction is $u_{k}^{\left(s\right)}=r_{k}^{\left(s\right)}/l_{k}^{\left(s\right)}$, and for each potential obstacle mesh, its axis-aligned bounding box $\left[B_{\min},B_{\max}\right]$ is first constructed. Meshes that cannot intersect with the ray are quickly eliminated using analytical ray-bounding box intersection criteria. For obstacles where the ray passes through the bounding box, the Möller–Trumbore ray–triangle intersection algorithm is applied to each of its triangular faces. If an intersection point $x\left(t^{*}\right)$ satisfies $0<t^{*}<d_{k}^{\left(s\right)}$, the candidate point is considered to be occluded at position s and cannot be used as an ERS point.

#### 3.2.3. Feasible Reference Point Set

Based on the combined results of instrument field-of-view and angle constraints and measurement interference checks, for each measurement station s, an initial reference point set $\Omega^{\left(s\right)}$ that satisfies the measurability constraints can be selected from the candidate reference point set Ω:

(59) $$\Omega^{\left(s\right)}=\left\{\left(P_{k},ID_{k}\right)\in \Omega |\alpha_{k}^{\left(s\right)}\in \left[45^{\circ },135^{\circ }\right]\right\}$$

Meanwhile, there is no ray obstruction between $\Omega^{\left(s\right)}$ and the measurement station s. It is worth noting that the optional sets $\Omega^{\left(s\right)}$ of different stations usually have intersections, which means that some ERS points on the tooling can serve multiple stations, which meets the actual needs of multi-station collaborative measurement in engineering.

### 3.3. Weighted Genetic Search Strategy for ERS Point Layout Based on BP Surrogate Model

After constructing the initial baseline set $\Omega^{\left(s\right)}$, it is still necessary to select a small number of baselines from it to form the final ERS set so that the transformation sensitivity index S is as small as possible while maintaining a reasonable spatial layout. Directly performing a combinatorial search on the analytical sensitivity model is computationally intensive; therefore, this paper introduces the BP surrogate model constructed in Section 2 to accelerate the sensitivity assessment process to the microsecond level, making large-scale evolutionary search possible.

#### 3.3.1. Optimization Objectives and Engineering Constraints

Given a measurement station s and the number of ERS points N, assuming the ERS point layout scheme consists of a subset $\varepsilon^{\left(s\right)}=\left\{P_{1}^{\left(s\right)},\ldots ,P_{N}^{\left(S\right)}\right\}$ selected from the initial reference point set $\Omega^{\left(s\right)}$, this paper considers the following three main evaluation indicators:

1. Comprehensive Sensitivity Index:

Using the BP surrogate model, with the distance, azimuth, and zenith angle $\left(d_{i},\phi_{i},\alpha_{i}\right)$ of the ERS point relative to the measurement station as input, the sensitivity components of the transformation parameters and the comprehensive index S are rapidly predicted. The smaller the comprehensive index S, the weaker the amplification effect of the transformation matrix on observation noise, and the more stable the overall coordinate transformation. It is the primary measure of the quality of the ERS layout.

2. Uniformity Index:

Let D be the distance matrix formed by the pairwise Euclidean distances within the ERS point set. For each point take its nearest neighbor distance $d_{i}^{NN}$ is taken with other points. The uniformity index is defined as:

(60) $$U=\frac{std\left(d_{1}^{NN},\ldots ,d_{N}^{NN}\right)}{mean\left(d_{1}^{NN},\ldots ,d_{N}^{NN}\right)}$$

The smaller U is, the more uniformly the ERS points are distributed in space, which is beneficial for improving the geometric condition number of the rigid body small transformation model and reducing the degradation of sensitivity due to local clustering.

3. Distance Constraint Penalty:

To avoid the accumulation and amplification of ranging noise caused by ERS points being too far from the measurement station and also to avoid insufficient geometric leverage due to points being too close, this paper constrains the average distance from ERS points to the measurement station using a smooth distance penalty function:

(61) $$D_{mean}=\frac{1}{N}\sum_{i=1}^{N}d_{i}$$

A penalty term $\exp\left[\left(D_{mean}/D_{0}^{2}\right)\right]$ is constructed such that when the average distance is significantly greater than the engineering reference value $D_{0}$, the fitness significantly deteriorates. This term is not a hard physical limitation of the instrument but rather reflects the engineering preference of “preferring relatively closer ERS points in large-scale measurements”.

Based on the above three indicators, this paper encodes the merits of ERS layouts into a weighted fitness function:

(62) $$F=\omega_{S}\tilde{S}+\omega_{U}\tilde{U}+\omega_{D}\tilde{D}$$

where $\tilde{S},\tilde{U},\tilde{D}$ represent the sensitivity, uniformity, and distance indices after dimensional normalization, and $\omega_{S},\omega_{U},\omega_{D}$ represent the weighting coefficients. The weighting coefficients are set to $\omega_{S}=0.5,\omega_{U}=0.3,\omega_{D}=0.2$. These values were determined through a sensitivity analysis to establish a priority hierarchy: precision ($\omega_{S}$) > spatial distribution ($\omega_{U}$) > distance ($\omega_{D}$). Sensitivity analysis indicates that while increasing $\omega_{U}$ (e.g., >0.6) yields a more geometrically regular layout, it forces the selection of points with suboptimal viewing angles, resulting in a degradation of the transformation accuracy (*S* increases by approx. 15~20%). Therefore, the current configuration ensures that physical precision remains the dominant optimization objective, with uniformity serving as a regularization term to prevent local clustering.

Considering that ERS are mostly arranged on the ground or on tooling and that some assembly tooling is not a full-envelope configuration, it is difficult for the envelope range of ERS points to completely enclose the target measurement point. Therefore, this paper uses the envelope volume as an auxiliary evaluation index, which is not included in the optimization objective but provides an additional reference when comparing multiple candidate schemes. It is used as an auxiliary tie-breaker when multiple schemes have comparable fitness.

#### 3.3.2. Genetic Search Process and BP Acceleration Strategy

Given the number of measurement stations s and the number of ERS points N, this paper employs an evolutionary genetic search algorithm based on a weighted fitness function to find the approximate optimal layout of ERS points. The main steps are as follows:

1. Initial population generation:

In the candidate reference point set $\Omega^{\left(s\right)}$ of station s, an initial population of size P is randomly generated, and each individual corresponds to an ERS point combination $\varepsilon^{\left(s\right)}$ consisting of N candidate reference point indices.

2. Batch sensitivity assessment of BP proxy model:

For each individual in the population, the $\left(d_{i},\phi_{i},\alpha_{i}\right)$ values of the ERS point relative to the measurement station s are sorted from largest to smallest by distance and azimuth and concatenated into an input vector. After a standardization transformation that is completely consistent with the training stage in Section 2, the vector is input into the corresponding BP neural network N to predict the sensitivity results, and the comprehensive sensitivity prediction value SS of each individual is obtained.

3. Fitness calculation and selection:

Based on the obtained $\hat{S}$, the fitness F of each individual is calculated by combining the nearest neighbor distance and average station distance of the ERS point set. Then, the next-generation parent individuals are selected from the individuals with better fitness.

4. Mutation and recombination:

To maintain population diversity, this paper employs a mutation operator primarily based on single-point mutation: for selected parent individuals, one of the baseline point indices is randomly replaced with a candidate point index that has not yet appeared, with a certain probability, while ensuring that indices within the individual are not duplicated. This operation avoids the design of complex crossover operators and can locally explore the ERS point layout while maintaining a relatively optimal structure.

5. Iterative updates and convergence criteria:

The evaluation–selection–mutation process is repeated for several generations until a preset number of iterations is reached or the fitness improvement is less than a certain threshold. For each N, the best sensitivity value ${\hat{S}}_{\min}^{\left(N\right)}$ and the corresponding ERS point layout scheme $\varepsilon_{best}^{\left(S\right)}\left(N\right)$ that has appeared throughout the entire evolutionary process are recorded.

### 3.4. Global Decision Mechanism for Adaptive Number of Points

The aforementioned genetic search yields a set of approximately optimal ERS point layout schemes $\varepsilon_{best}^{\left(S\right)}\left(N\right)$ and the corresponding comprehensive sensitivity index. ${\hat{S}}_{\min}^{\left(N\right)}$ for each number of ERS points N. However, in practical engineering applications, the number of ERS points not only affects the cost of point layout and construction and the complexity of on-site maintenance but may also impact the workload of subsequent coordinate system establishment and reference point drift monitoring. Therefore, it cannot be simply assumed that a larger N is always better; a systematic trade-off needs to be struck between sensitivity and the number of ERS points.

To address this, this paper introduces a point-adaptive global decision-making mechanism. The specific approach is as follows:

1. Find the minimum sensitivity baseline across all N:
  (63) $$S_{\min}=\underset{N}{\min}{\hat{S}}_{\min}^{\left(N\right)}$$
2. Set a tolerance coefficient $\eta \left(5\%\right)$. Identify the candidate set where:
  (64) $${\hat{S}}_{\min}^{\left(N\right)}\leq S_{\min}\left(1+\eta \right)$$
  All ERS point schemes are considered as a candidate set for “sensitivity compliance”.
3. Select the scheme with the minimum N from the qualified set as the final ERS layout:
  (65) $$N^{*}=\arg\underset{N:{\hat{S}}_{\min}^{\left(N\right)}\leq S_{\min}\left(1+\eta \right)}{\min}N$$
  (66) $$\varepsilon_{final}^{\left(s\right)}=\varepsilon_{best}^{\left(s\right)}\left(N^{*}\right)$$

This strategy intuitively embodies the engineering philosophy of “reducing the number of ERS points as much as possible without significantly sacrificing sensitivity performance” to improve measurement efficiency and avoid unnecessary reference point redundancy.

## 4. Experiments

### 4.1. Experimental Scenario and Parameter Configuration

**Figure 12:**
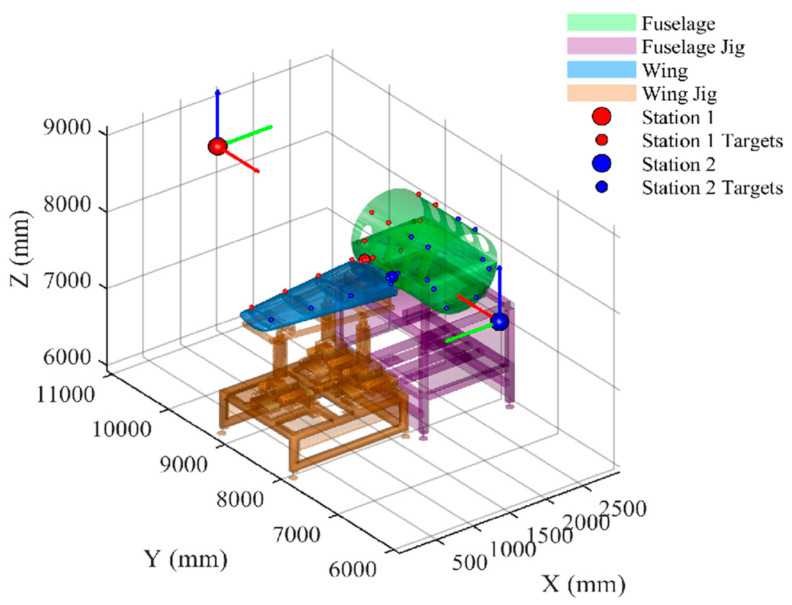
3D simulation environment setup for fuselage and wing assembly measurement.

The experimental setup included fuselage sections, wing sections, and their corresponding assembly fixtures, as shown in Figure 12. Two laser tracker stations (Station 1 and Station 2) and their respective matched target test points were deployed on-site.

**Figure 13:**
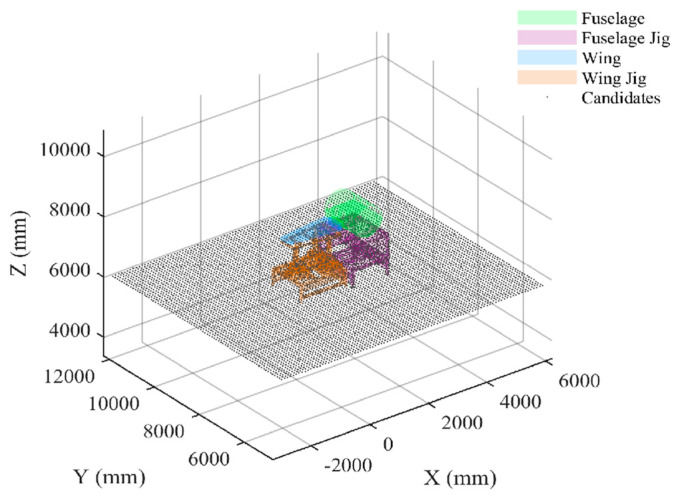
Visualization of generated candidate ERS points on ground and tooling surfaces.

Based on the method in Section 3.1, the ground and tooling surfaces are discretized in this scenario to generate a candidate reference point set Ω, as shown in Figure 13, with a total of 9252 candidate ERS points generated.

Following the selection strategy outlined in Section 3.2, feasibility checks were performed on both stations to obtain the available baseline points $\Omega^{\left(s\right)}$. After selection, Station 1 retained 3619 available baseline points, and Station 2 retained 3852 available baseline points.

In the intelligent optimization phase, the genetic algorithm adopted the following typical parameter settings:

1. Population size = 50,000 to improve coverage in the high-dimensional combinatorial space;
2. Generations = 100 to ensure search quality while considering computation time;
3. The relative tolerance coefficient η=5%, where η controls the trade-off between the number of reference points *N* and the sensitivity *S*, and it can be adjusted according to tolerance requirements.

### 4.2. ERS Point Layout Planning Results

As the population size and generation number increase, the search accuracy of the ERS point layout improves, but the computational workload also increases, making program runtime comparisons less meaningful. Under the parameters set in this paper, the program runtime was 26 min. ERS point layout optimization was performed on two measurement stations within the point count range N = 4~15, and the results are as follows:

1. Station 1: When *N* = 4, the optimal sensitivity ${\hat{S}}_{\min}^{\left(4\right)}$ is approximately 4.9958 × 10^−4^. As *N* increases, ${\hat{S}}_{\min}^{\left(N\right)}$ decreases monotonically, reaching a minimum value of ${\hat{S}}_{\min}^{\left(14\right)}=6.2152\times 10^{-5}$ at *N* = 14. At a tolerance of 5%, although *N* = 15 slightly increases, it does not meet the tolerance threshold. Therefore, $N^{*}=14$.
2. Station 2: Similarly, ${\hat{S}}_{\min}^{\left(N\right)}$ decreases overall as *N* increases, reaching a minimum of ${\hat{S}}_{\min}^{\left(14\right)}=6.4513\times 10^{-5}$ at *N* = 14, and it rebounds slightly at *N* = 15. With a 5% tolerance, only *N* = 14 still meets the conditions, so $N^{*}=14$ is selected for the global decision.

The final planned ERS point coordinates are shown in Table 4, including ground points, fuselage tooling points, and wing tooling points.

**Figure 14:**
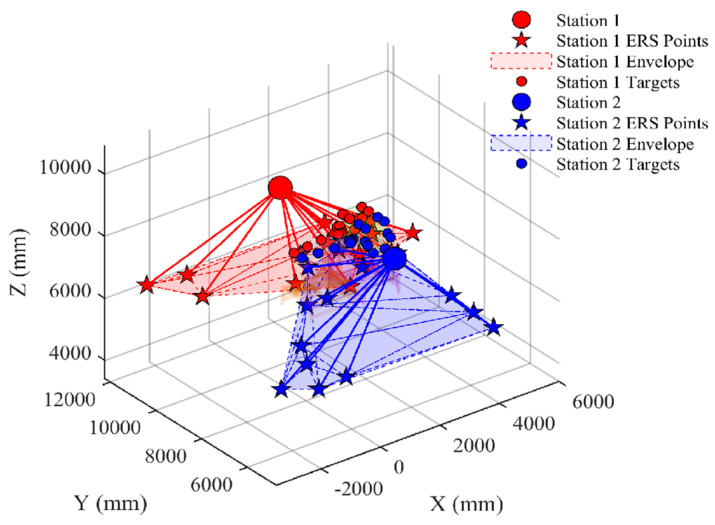
Optimized topological layout of ERS points for dual-station measurement.

The final optimized layout of the two stations is shown in Figure 14. It can be seen that the selected ERS points make good use of the advantages of the wide baseline of the ground and the vertical support of the tooling, forming a robust three-dimensional envelope around the measurement space.

Geometrically, neither of the two measurement stations’ respective ERS layouts forms a strict “convex hull” enclosing all target points. Instead, they combine with the ground and tooling to form a set of engineering-feasible three-dimensional support structures, which is consistent with the constraint in actual assembly workshops that “ERS points are placed in accessible and maintainable areas.” Sensitivity results show that, under the aforementioned geometric and engineering constraints, the weighted GA method driven by BP proxy can still compress the transformation sensitivity to the order of 10^−5^.

### 4.3. ERS Point Planning Result Accuracy Verification and Performance Analysis

To quantitatively evaluate the impact of the planned ERS point layout on the actual measurement accuracy of the target measurement points, this paper conducts 1000 Monte Carlo simulations at two measurement stations. Each simulation includes the following:

Theoretical baseline error: Ignoring coordinate system registration and considering only the measurement error caused by instrument noise, the RMS residual under the instrument’s inherent error is obtained, denoted as $RMS_{Baseline}$.

Actual transformation error: Introducing instrument uncertainty, the planned ERS points are measured to complete the registration between the instrument coordinate system and the assembly coordinate system. The target point to be measured is measured to obtain the RMS residual including the registration error, denoted as $RMS_{Actual}$.

Error increment introduced by registration: The difference between the measured transformation error and the theoretical reference error is defined as the Registration-Induced Error (RIE), which reflects the additional error introduced by coordinate transformation.

(67) $$RIE=RMS_{Actual}-RMS_{Baseline}$$

Relative loss introduced by registration: The ratio between RIE and $RMS_{Baseline}$ is defined as the Registration-Induced Loss Ratio (RILR), which reflects the proportion of accuracy loss caused by coordinate transformation.

(68) $$RILR=\frac{\Delta RMS_{reg}}{RMS_{\mathrm{Baseline}}}$$

#### 4.3.1. Global Statistical Performance Analysis

**Figure 15:**
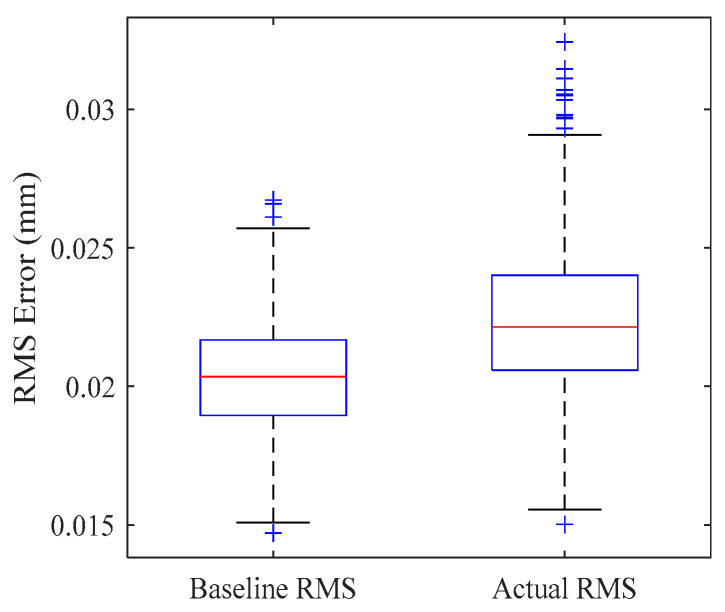
Station 1 RMS comparison.

**Figure 16:**
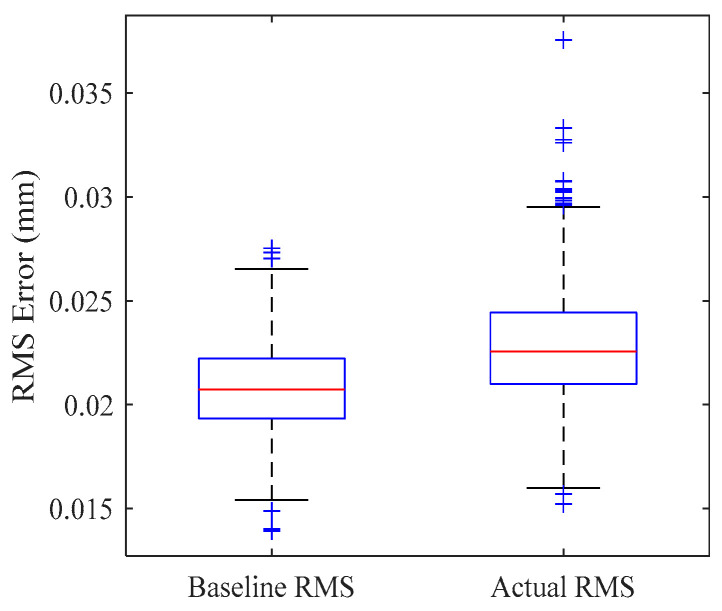
Station 2 RMS comparison.

Figure 15 and Figure 16 present the boxplots of the global RMS error distribution across 1000 simulations. It can be observed that for both Station 1 and Station 2, the distribution of the “actual RMS” aligns closely with the “baseline RMS” with only a marginal shift in the median values. This high degree of overlap indicates that the optimized ERS layout possesses excellent robustness, effectively suppressing the amplification of measurement noise during the computation of transformation parameters.

Table 5 summarizes the statistical mean results from the 1000 simulations. The data demonstrates that the optimized layouts achieve exceptional registration accuracy at both stations. Specifically, the RIE for Station 1 and Station 2 is controlled at 0.0019 mm and 0.0021 mm, respectively, with corresponding RILR values of only 9.31% and 10.04%. This implies that the additional uncertainty introduced by the coordinate registration step is merely around one-tenth of the instrument’s intrinsic error, fully satisfying the stringent engineering requirements of large-scale metrology.

#### 4.3.2. Point-Wise Accuracy and Uniformity Analysis

**Figure 17:**
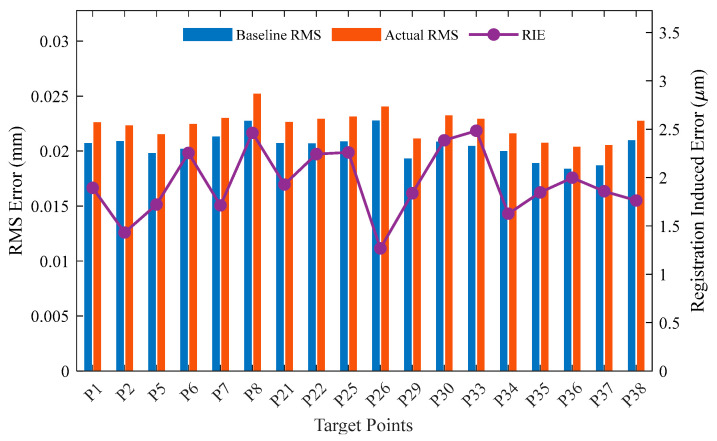
Point-wise breakdown of RMS error, RIE, and RILR for Station 1.

**Figure 18:**
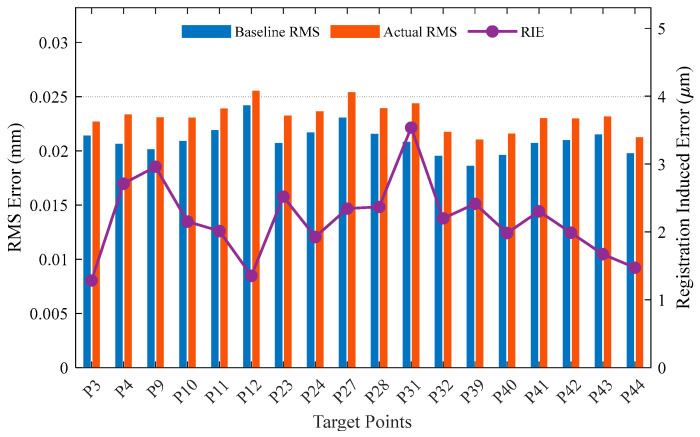
Point-wise breakdown of RMS error, RIE, and RILR for Station 2.

To further investigate the spatial distribution characteristics of the error, Figure 17 and Figure 18 illustrate the point-wise RMS error for each target test point.

Station 1 (Figure 17): The actual RMS values for all target points are uniformly distributed between 0.021 and 0.025 mm. The RIE, represented by the purple line, remains stable across all points, fluctuating only within the narrow range of 1.5~2.5 μm. No significant local regions of accuracy degradation are observed.

Station 2 (Figure 18): The results show a similar pattern. Although Station 2 has a wider coverage area, the RIE at each point is still stably controlled below 4 μm. This verifies that the benchmark layout planning method proposed in this paper not only ensures globally optimal sensitivity when dealing with complex occlusion constraints but also takes into account the spatial consistency of the measurement field accuracy.

#### 4.3.3. Comparison with Feasible Uniform Baseline Strategy

To rigorously evaluate the performance advantage of the proposed physics-based planning strategy over traditional geometric intuition, a comparative experiment was conducted. Since a theoretical grid distribution in 3D space is often practically infeasible due to the lack of physical support (e.g., points cannot be suspended in mid-air), a “feasible uniform baseline” was constructed. This baseline was generated by performing K-means clustering on the dense candidate points within the valid ground and tooling regions to select *N* = 14 representative points. This approach simulates a high-quality manual layout strategy that maximizes spatial uniformity subject to installation constraints.

The comparative verification results for both Station 1 and Station 2 are presented in Table 6. While the uniform baseline provides extensive spatial coverage, the proposed optimized layout significantly outperforms it in terms of measurement accuracy. Specifically, for Station 1, the Registration-Induced Error (RIE) decreased from 0.00330 mm (uniform baseline) to 0.00203 mm (optimized), representing an improvement of 38.43%, and the sensitivity *S* improved by 10.04%. Similarly, for Station 2, the RIE was reduced by 39.15%, and the sensitivity *S* improved by 2.91%.

These results demonstrate that relying solely on geometric uniformity is insufficient for high-precision metrology, as it fails to account for the anisotropic nature of laser tracker uncertainty. In contrast, the proposed method, driven by the physics-based sensitivity model, effectively identifies robust configurations that minimize error propagation, thereby validating its superiority over intuitive geometric planning.

## 5. Discussion

### 5.1. Efficiency vs. Precision Trade-Off

The proposed method effectively resolves the conflict between computational efficiency and registration accuracy in large-scale ERS point planning. By substituting the rigorous GUM analytical process with the BP neural network surrogate model, the sensitivity evaluation time for a single configuration is reduced from seconds to milliseconds (acceleration ratio > 37). This capability allows the genetic algorithm to explore a vast solution space within minutes, making dynamic re-planning feasible for digital twin applications—a capability that traditional analytical methods lack.

### 5.2. Significance of RIE and RILR Metrics

The introduction of the Registration-Induced Error (RIE) and Registration-Induced Loss Ratio (RILR) provides a quantitative standard for evaluating layout quality. Experimental results show that the RILR is maintained at approximately 10% (RIE ≈ 0.002 mm). This indicates that the additional uncertainty introduced by the optimized ERS layout is an order of magnitude lower than the instrument’s intrinsic noise. From a metrology perspective, this “10% loss” is an acceptable cost for achieving automation and avoiding occlusion. More importantly, the value of this cost becomes evident when compared to the “feasible uniform baseline.” As demonstrated in Section 4.3, the optimized layout reduces the RIE by approximately 40% compared to the uniform distribution strategy. This indicates that the proposed physics-based sensitivity optimization provides a significantly higher “return on precision” than intuitive geometric planning, effectively minimizing the error propagation risk in constrained spaces.

### 5.3. Adaptive Decision and Future Absolute Thresholds

The relative tolerance coefficient η (set to 5%) successfully identified a “knee point” at *N* = 14. This parameter serves as a tunable lever: a higher η prioritizes efficiency (fewer points), while a lower η pursues theoretical precision. Currently, the optimization relies on the relative sensitivity index (*S*). However, since the current method focuses on finding the optimal geometric solution, the resulting layout is not necessarily regularly distributed. Future work will evolve towards collaborative optimization with tooling. By propagating the sensitivity to the target points to calculate the absolute measurement uncertainty, the system could stop increasing *N* once the absolute uncertainty meets a specific threshold (e.g., *U* < 0.05 mm). This would allow for a more regularized distribution of points guided by tooling design constraints, achieving a tighter integration of metrology and assembly resources.

### 5.4. Generalization and Deployment Workflow

Addressing practical deployment concerns regarding instrument changes and new assembly tasks, the generalization capability of the proposed method is defined by two levels: the surrogate model level and the planning strategy level.

#### 5.4.1. Instrument Adaptability

The BP neural network essentially learns the error propagation mechanism determined by the instrument’s intrinsic uncertainty parameters (Equations (3) and (4)). Therefore, retraining is required only when the measurement hardware model changes. For the same instrument model, the trained BP network is universally applicable across any spatial measurement scenario without retraining.

#### 5.4.2. Scene Adaptability

The weighted genetic algorithm relies on specific geometric constraints (obstacles, target distribution). Consequently, for recurrent assembly of the same aircraft model, the optimization is performed once and the layout is reused. However, for new aircraft models or new assembly steps (where CAD models and target points change), the optimization process needs to be re-executed. Thanks to the millisecond-level inference speed of the BP surrogate model, this “re-planning” process is highly efficient (typically completing within minutes), making it compatible with flexible manufacturing requirements.

In summary, the workflow for a new part involves: (1) inputting the new CAD model; (2) loading the pre-trained BP model (instrument-specific); (3) running the feasibility check and GA optimization (scene-specific); and (4) outputting the new ERS layout.

## 6. Conclusions

Addressing the issues of low efficiency, susceptibility to occlusion, and reliance on manual experience in the layout of Enhanced Reference System (ERS) points for large-scale aircraft component assembly measurement fields, this paper proposes an adaptive planning method combining physical mechanisms and data-driven models. The main research conclusions are as follows:

(1) Methodological Innovation: A registration sensitivity surrogate model was constructed by fusing GUM uncertainty theory with deep learning. The model achieves high-fidelity prediction ($R^{2}$ 0.99, error < 4%) while boosting computational speed by orders of magnitude, enabling real-time optimization.
(2) Intelligent Optimization: A weighted genetic algorithm incorporating ray-tracing occlusion detection was developed. The strategy effectively leverages both ground and tooling resources to construct a valid measurement network, ensuring 100% visibility of target points.
(3) Quantitative Performance: An adaptive decision mechanism identified the optimal layout with 14 points. Verification shows that the Registration-Induced Error (RIE) is controlled at approximately 0.002 mm, and the Registration-Induced Loss Ratio (RILR) is maintained at about 10%. Crucially, comparative verification reveals that the RIE is reduced by approximately 40% compared to the feasible uniform baseline. This confirms that the proposed physics-based data-driven method yields superior accuracy over geometric experience while strictly meeting high-precision assembly requirements.

In summary, this method provides a theoretical foundation and a practical tool for the automated construction of digital twin measurement fields, ensuring high-precision coordinate traceability in intelligent aircraft assembly.

## Figures and Tables

**Table 1:** Statistical performance metrics of the BP neural network model.

N	Train_R	Val_R	Test_R	All_R	MSE
4	0.975458	0.974404	0.973451	0.975781	2.366241 × 10^−3^
5	0.986520	0.985460	0.985277	0.986175	1.568127 × 10^−3^
6	0.991319	0.989854	0.990190	0.990922	1.471129 × 10^−3^
7	0.989567	0.988292	0.988444	0.989216	1.457522 × 10^−3^
8	0.988935	0.987298	0.987729	0.988517	1.523793 × 10^−3^
9	0.992536	0.991622	0.991634	0.992256	1.274588 × 10^−3^
10	0.991924	0.989068	0.989099	0.991073	1.409922 × 10^−3^
11	0.993334	0.992273	0.992194	0.993375	1.126105 × 10^−3^
12	0.993156	0.992492	0.992484	0.992956	1.148843 × 10^−3^
13	0.993626	0.992360	0.992015	0.993188	1.190548 × 10^−3^
14	0.992682	0.991399	0.991152	0.992252	1.173369 × 10^−3^
15	0.991619	0.990444	0.989882	0.991185	1.137516 × 10^−3^

**Table 2:** Validation results of prediction accuracy on independent test sets.

N	R^2^_Log	RMSE_Log	MAE_Phys	MRE_Phys	P95_Phys
4	0.9661	0.0713	1.42 × 10^−4^	0.0802	5.21 × 10^−4^
5	0.9804	0.0461	4.33 × 10^−5^	0.0543	1.64 × 10^−4^
6	0.9874	0.0341	2.34 × 10^−5^	0.0445	7.73 × 10^−5^
7	0.9856	0.0339	1.65 × 10^−5^	0.0431	5.55 × 10^−5^
8	0.9887	0.0281	1.16 × 10^−5^	0.0402	3.37 × 10^−5^
9	0.9903	0.0250	8.24 × 10^−6^	0.0333	2.44 × 10^−5^
10	0.9900	0.0233	6.23 × 10^−6^	0.0305	1.94 × 10^−5^
11	0.9928	0.0191	4.50 × 10^−6^	0.0264	1.28 × 10^−5^
12	0.9929	0.0189	4.11 × 10^−6^	0.0274	1.09 × 10^−5^
13	0.9931	0.0173	3.33 × 10^−6^	0.0246	9.13 × 10^−6^
14	0.9923	0.0175	3.04 × 10^−6^	0.0252	7.98 × 10^−6^
15	0.9921	0.0179	2.87 × 10^−6^	0.0252	8.45 × 10^−6^

**Table 3:** Computational time comparison: GUM vs. BP neural network surrogate model.

M	GM (Seconds)	BP (Seconds)	Ratio
100	0.0123	0.0122	1.01
1000	0.082	0.0129	6.36
10,000	0.7748	0.0317	24.47
100,000	7.8534	0.2107	37.27

**Table 4:** Optimized ERS point layout coordinates for Station 1 and Station 2.

Station 1				Station 2			
ID	x	*y*	*z*	ID	*x*	*y*	*z*
GND_1670	−899.91	12,041	5929.9	GND_1620	−899.91	7041	5929.9
GND_1354	−1299.9	10,841	5929.9	WG_JIG_387	650.14	7920.2	6517.6
GND_760	−2099.9	12,241	5929.9	GND_5255	3900.1	5741	5929.9
GND_5996	4800.1	11,441	5929.9	GND_2777	600.09	8741	5929.9
FG_JIG_65	1734.1	8315.3	6345.3	GND_5169	3800.1	4741	5929.9
GND_5243	3800.1	12,141	5929.9	GND_1523	−999.91	4941	5929.9
WG_JIG_310	460.6	8851.7	7571.5	GND_762	−1999.9	4841	5929.9
GND_5523	4200.1	9741	5929.9	WG_JIG_212	428.71	8478.9	7340.9
GND_6364	5300.1	10,241	5929.9	GND_5342	4000.1	6841	5929.9
FG _JIG_330	1902.8	8159	7401.7	FG _JIG_999	2878.7	7748.9	7279.9
WG_JIG_381	574.75	9199.9	6438.1	GND_160	−2799.9	5441	5929.9
FG _JIG_124	2957	8968.2	7301.7	FG _JIG_109	1736.1	8823.6	7282.9
FG _JIG_161	1739.2	9302	7301.7	FG _JIG_28	1736.1	7804.2	7190
FG _JIG_1112	2906.1	9284	6183.6	GND_1231	−1399.9	6141	5929.9

**Table 5:** Global accuracy metrics: statistical mean of 1000 Monte Carlo simulations.

Station Name	Baseline RMS (mm)	Actual RMS (mm)	Average Maximum Error (mm)	RIE (mm)	RILR
Station 1	0.0204	0.0223	0.0383	0.0019	9.31%
Station 2	0.0209	0.0230	0.0397	0.0021	10.04%

**Table 6:** Comparison of verification results between feasible uniform baseline and optimized layout (*N* = 14).

Station	Layout	*S*	Baseline RMS (mm)	Actual RMS (mm)	RIE (mm)	RILR
Station 1	Uniform Baseline	6.9091 × 10^−5^	0.02037	0.02367	0.00330	16.19%
	Optimized	6.2152 × 10^−5^	0.02036	0.02239	0.00203	9.97%
Station 2	Uniform Baseline	6.6444 × 10^−5^	0.02090	0.02427	0.00337	16.10%
	Optimized	6.4513 × 10^−5^	0.02084	0.02289	0.00205	9.83%

## Data Availability

The original contributions presented in this study are included in the article. Further inquiries can be directed to the corresponding author.
